# Retroperitoneal laparoscopic technique in treatment of complex renal stones: 75 cases

**DOI:** 10.1186/1471-2490-14-16

**Published:** 2014-02-04

**Authors:** Chao Qin, Shangqian Wang, Pu Li, Qiang Cao, Pengfei Shao, Pengchao Li, Zhijian Han, Jun Tao, Xiaoxin Meng, Xiaobing Ju, Rijin Song, Jie Li, Wei Zhang, Qiang Lu, Changjun Yin

**Affiliations:** 1 Department of Urology, the First Affiliated Hospital of Nanjing Medical University, Nanjing, 300 Guangzhou Road, Nanjing, Jiangsu 210029, China

**Keywords:** Retroperitoneal laparoscopic technique, Complex renal stones, Ureteroscope, Treatment

## Abstract

**Background:**

In most hospitals, several options for the management of renal stones are available: shockwave lithotripsy, endourologic treatment, or surgery. Choice of treatment is based on the anatomic characteristics of the patient, and the location and size of the stones. In this study we assessed a retroperitoneal laparoscopic technique for treatment of complex renal stones.

**Methods:**

Seventy-five patients, including 53 men and 22 women with a mean age of 47.8 years (range 18–74 y), underwent retroperitoneal laparoscopy for the treatment of complex renal stones between July 2006 and November 2012 in our hospital.

**Results:**

The retroperitoneal laparoscopic procedures for treatment of complex renal stones were completely successful in 73 cases, while 2 cases converted to open surgery. The operative time was 85–190 min with a mean of 96 min. The estimated blood lost was 20–400 mL with a mean of 80 mL. After the operation 7 patients experienced urinary leakage. Ultrasonography, x-ray of the kidney, ureter and bladder, and intravenous urography were reviewed at post-procedural follow-up at 6–82 months. No hydronephrosis aggravation was found, and there was no calculus recurrence.

**Conclusion:**

The merits of retroperitoneal laparoscopy for the treatment of complex renal stones include sparing the nephron, less bleeding, short hospitalization, quick postoperative recovery, and controllable procedure after training Success depends on the experience of surgeons and judicious selection of cases.

## Background

Shockwave lithotripsy or endourologic treatments such as percutaneous nephrostolithotomy are the primary options in most cases of renal stones. Open and laparoscopic procedures are also applied for some selected patients. In complex situations such as abnormal anatomy, accompanying complications, or larger stones, surgery is often the primary therapeutic option.

Retroperitoneal laparoscopy for renal surgery is a viable and versatile alternative to transperitoneal access; the location of the kidneys in the retroperitoneum makes it a perfect approach. Moreover, due to low postoperative complications, reduced hospitalization, less blood loss, and better cosmetic results, laparoscopy has prevailed in recent years. Nevertheless, only in centers with adequate experience in transperitoneal and retroperitoneal laparoscopic procedures should these cases be performed laparoscopically [1].

Retroperitoneal laparoscopy is a minimally invasive approach, compared to endourologic treatment, for a variety of reconstructive indications for different pathologic conditions. After adequate training, surgeons should be able to use the approach proficiently. Despite the limited surgical space, direct posterior access to the kidney and renal hilum makes this attractive, as it allows early ligation of renal vessels. Emerging techniques such as single port or single incision could also be performed in a selected subset of patients. The general acceptance of this technique worldwide is confirmation of its potential value.

In the present study, we report the outcomes of 75 patients who underwent retroperitoneal laparoscopic pyelolithotomy for the treatment of complex renal stones between July 2006 and December 2012 in our hospital.

## Methods

### Patients

We chose 75 patients including 53 men and 22 women with a mean age of 47.8 years (range 18–74 years) as research subjects (Table 1). Any of the following were criteria for inclusion in the study: 1) a renal pelvic stone with complications, such as nephroptosis, ureteropelvic junction obstruction (UPJO), or retrocaval ureter; 2) a relatively large non-staghorn solitary stone in the renal pelvis not suitable for extracorporeal shockwave lithotripsy or ureteroscopic lithotripsy, or treatment failure cases; or 3) multiple renal stones in the pelvis and calyx without obvious hydronephrosis, especially calyceal stones in the former, which are relatively difficult for percutaneous nephrostolithotomy.

**Table 1:** Patient demographics and clinicopathologic features

Age (y)	
Mean	47.8
Range	18-74
Gender	
Male	30 (71.4)*
Female	12 (28.6)
Stone size (cm)	
Mean	2.1
Range	1.5-3.5
Stone location	
Left	28 (66.7)
Right	14 (33.3)
Symptoms	
Renal colic	30 (71.4)
No symptoms	12 (28.6)
Hydronephrosis	
Mild to moderate	32 (76.2)
Severe	10 (23.8)
Complications	
Nephroptosis	3 (7.1)
UPJO	8 (19.0)
Retrocaval ureter	1 (2.4)
Aberrant crossing vessels	1 (2.4)

*Data in parentheses are percentages.

All the patients were examined with ultrasonography, intravenous urogram, and computed tomography to assess the renal collecting system anatomy and characteristics of stones, including size and location. Retrograde urography was performed for patients with suspect UPJO.

### Procedures

All the procedures were performed in accordance with the ethical standards of the committee on human experimentation of the Nanjing Medical University. And written informed consent for participation in the study was obtained from participants.

General anesthesia was used for all patients. Patients were positioned in the lateral decubitus position with hyperextension. A 1.5-cm incision was made in the mid-axillary line 1 cm above the iliac crest. Hemostatic forceps were used to divide the fascia lumbodorsalis. The retroperitoneal space was separated by digital Dissection. A working space was created in the retroperitoneum by self-made balloon dilation with 300–500 mL of air for 5 minutes. Three ports were guided by index finger and placed at the anterior axillary line (5-mm port), the subcostal posterior axillary line (12-mm port), and the previous mid-axillary line (10-mm port). Trocars were then inserted and artificial pneumoperitoneum was created by CO_2_ insufflation (1.6-2.0 kPa). A 30° telescope was introduced through the port.

For obese patients, too much fat in the retroperitoneum can limit the surgical field influenced by respiration-induced peritoneal movement. In such patients, the operation may be completed successfully with the help of a 5-mm fourth port, inserted 3–4 cm anterior to the first port. This additional port could also help deal with UPJO (ligation and reconstruction), intrasinusal pyelolithotomy and the other cases when the forth port may help seperate or reconstruction.

### Laparoscopic pyelolithotomy combined with flexible ureteroscopy

Location of the psoas muscle as a marker helps to remove extraperitoneal fat. For extra-renal pelvic stones, the dorsal part of the renal pelvis and ureter are identified. Acting gently can prevent extracted stones from slipping into calices. After touching the stone with forceps, a vertical incision is made and the stone is extracted. A catheter (F10) is inserted through the trocar and into the incision and irrigated by saline, whereby most of the residual stones can be flushed out. A double-J stent (F7) through the pelvic incision is positioned to the bladder.

**Figure 1:**
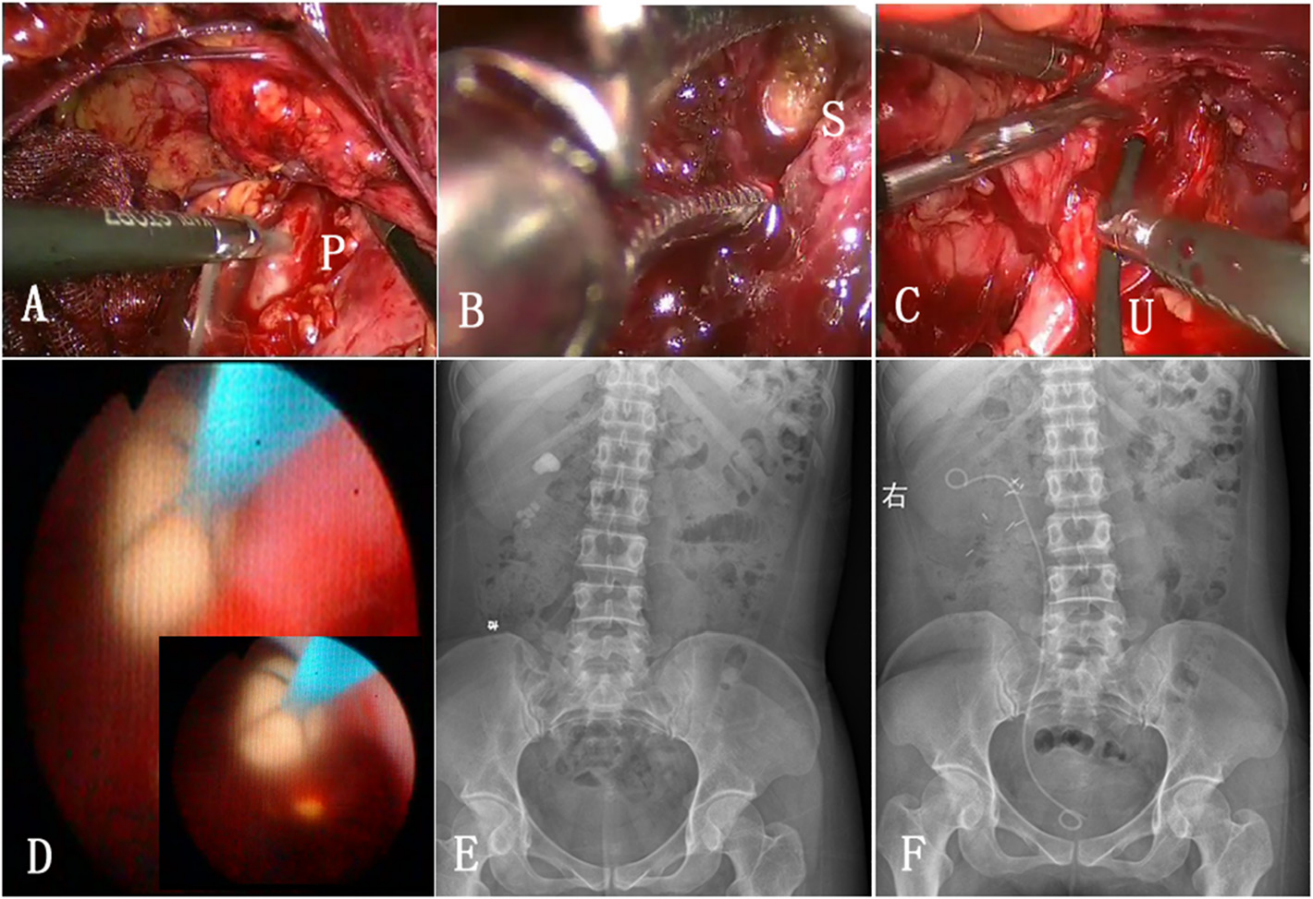
**Illustration for whole procedure of stone extraction. A**: The intrasinusal pelvis was incised according to the shape and location of the stone. P represents pelvis. **B**: The stone was made free from mucosal adhesions with forceps. S represents stone in the pelvis. **C**: A flexible ureteroscope was inserted from the trocar under the twelfth rib and through the pelvis incision to search for the residual stones. U represents ureterscope. **D**: A large stone was removed by stone basket. **E-F**: Intraoperative and postoperative abdominal x-ray was used to confirm stone clearance.

For patients with intra-renal pelvic or severe perirenal adhesions, the isolation of the renal sinus is relatively difficult. We first open Gerota’s fascia and find the inferior pole of the kidney, then reach the upper ureter along the surface of the psoas muscle and trace upwards to the pelvis. Intrasinus fat is removed along the outer membrane of the pelvis, up to the deep pelvis and initial portion of renal calices and infundibulum, for intra-renal stones in the pelvis. Attention must be paid as to whether there are aberrant vessels crossing the renal sinus. The intrasinusal pelvis is incised according to the shape and location of the stone (Figure 1A). The stone is freed from mucosal adhesions with forceps (Figure 1B). The remaining stones are flushed from pelvis and a double-J stent (F7) through the incision is positioned from the renal pelvis to the bladder. The pelvis incision is sutured using 5–0 Vicryl absorbable suture.

If residual stones in the renal calyx are likely, a flexible ureteroscope is inserted from the trocar under the twelfth rib and through the pelvis incision to search carefully for residual stones (Figure 1C). Large stones (>2 cm) could be crushed with a Holmium laser or removed by stone basket (Figure 1D), whereas small stones are flushed out directly. The intraoperative and postoperative abdominal x-ray was used to confirm stone clearance (Figure 1E-F).

Written informed consent was obtained from the patient for publication of any accompanying images. A copy of the written consent is available for review by the Editor of this journal.

### Solitary stone with complications

For patients with UPJO, we used a method previously described [2]. Briefly, stones were removed directly after pyelotomy and then ureteropyelostomy was performed. The ureter was first spatulated at the lateral border to a sufficient length, and then transected from the renal pelvis. UPJO can be repaired by a pelvis-to-ureter anastomosis.

For patients with a retrocaval ureter, the inferior vena cava was lifted with dissecting forceps and the ureter was mobilized in the interaortocaval region, where it passes posterior to the inferior vena cava. The proximal ureter, lateral to the inferior vena cava, was dissected up to the UPJ level. The ureter at the UPJ was transected and the atretic unhealthy portion (approximately 2 cm in length) was excised, after which the ureter was spatulated for 2 cm. A vertical incision in the pelvis was made and the stone was retrieved with the help of a grasper.

For patients with nephroptosis, after the stone was removed the kidney was fixed using two non-absorbable polyester sutures. The upper pole was fixed to the psoas muscle, and the convexity of the kidney was fixed to the dorsal abdominal wall. Both sutures passed through the renal parenchyma and were tied intracorporeally.

## Results

The retroperitoneal laparoscopic technique for treatment of complex renal stones was completely successful in 73 cases, while 2 cases were converted to open surgery (Table 2). The stone was difficult to remove in one case due to an intra-renal small pelvis, and in another due to severe perirenal inflammation caused by repeated extracorporeal shockwave lithotripsy. The stones were eliminated in 69 cases during operations, and residual stones were discharged by subsequent extracorporeal shockwave lithotripsy in 6 cases. Eight cases with UPJO received pyeloplasty. Three cases with nephroptosis received nephropexy. Two patients with retrocaval ureter and 2 with aberrant crossing vessels received ureteral chop amputation and reconstruction.

**Table 2:** Results

Operative time (min)	
Mean	96
Range	85-190
Blood loss (mL)	
Mean	80
Range	20-400
Laparoscopic intrasinusal pyelolithotomy, *n* (%)	16 (38.1)
Laparoscopic pyelolithotomy + ureteroscope, *n* (%)	13 (30.9)
Pelvis-ureter running anastomosis for UPJ, *n* (%)	8 (19.0)
Nephropexy for nephroptosis, *n* (%)	3 (7.1)
Ureter reconstruction for retrocaval ureter, *n* (%)	1 (2.4)
Chop amputation for aberrant crossing vessels, *n* (%)	1 (2.4)

No serious intraoperative complication occurred in any of the 75 cases. The retroperitoneal drainage tube was removed 3–6 days after the procedure, the Foley catheter at 8 days and the stitches at 7 days. Sixty-eight patients experienced no urinary leakage after the operation. In other cases with urinary leakage, the drainage tube was removed less than 3 weeks after surgery. The double-J stent was extracted in outpatient clinic 1 month later. Ultrasonography, x-ray of the kidney, ureter and bladder, and intravenous urography were reviewed during 6–82 months of follow-up and no aggravating hydronephrosis was found.

## Discussion

Since the introduction of shockwave lithotripsy in 1980 for the management of renal stones, minimally invasive therapy for urolithiasis has improved and various new techniques have been introduced [3]. Advances in extracorporeal shockwave lithotripsy and intracorporeal (transurethral or percutaneous) lithotripsy have changed the treatment of urinary stones [3]. Previous to these developments, surgical management was the only available option for urinary stones, whereas now there are many minimally invasive modalities to select from, such as percutaneous nephrolithotomy and ureteroscopy. Moreover, the introduction of different methods of stone fragmentation has improved the rate of stone clearance [4]. With regard to renal stones, percutaneous nephrostolithotomy is the treatment of choice in most cases.

Laparoscopic surgery via the retroperitoneal approach confers several advantages compared to the transperitoneal approach. Our clinical experience shows that retroperitoneal laparoscopic intrasinusal pyelolithotomy can be considered even when the stone is located in the intra-renal pelvis, with the exception of large staghorn renal stones. Many urologists have studied two different operative routes. Compared with the transperitoneal, the retroperitoneal approach decreases complications of injury to surrounding visceral organs, bowel paralysis, and adhesion. In addition, it is easy to obtained better exposure to the retroperitoneal anatomy.

Relevant anatomical research [5] shows renal sinus fat,enclosing the renal vessels, between the pelvis and parenchyma has a close relationship to renal parenchyma. A layer of connective tissue (the renal pelvis membrane) exists between the sinus fat and pelvis, whose blood supply is from the pelvic muscle layer but not from renal vessels in sinus fat. Therefore, the seperation of the intrasinusal pelvis along with the pelvis connective membrane does not cause bleeding. When the pelvis has extensive adhesions with the surrounding tissues as in inflammation, and the pelvis membrane adheres with sinus fat, the dissection of the membrane can be easy and the pelvis can be exposed without serious bleeding along this route.

Perirenal inflammation can be very serious for patients with long-term obstruction or repeated extracorporeal shockwave lithotripsy. We can first isolate the ureter at the lower pole of the kidney, and then determine the location of the pelvis. The surgeon must take extra care when clamping stones after the pelvis is opened, to avoid tearing the renal pelvis. The renal pelvis mucosa where the stones are located is brittle because of edema, so do not force the suture when tension is high. The incision can be covered by renal sinus fat to reduce the risk of postoperative urinary leakage.

In the present study, retroperitoneal laparoscopic intrasinusal pyelolithotomy was successful in most patients. This technique is minimally invasive and can surpass open surgery in merit, with no injury to the nephron, less bleeding, simple manipulation, short hospitalization, and quick postoperative recovery, without incision of the renal parenchyma.

We combined laparoscopic techniques with endourology to develop a method of extracting multiple pelvic calyceal stones. The flexible ureteroscope can be guided into the renal pelvis easily after the ureteropelvic junction is resected through the 10-mm port. Irrigant is collected concurrently with a suction device passed through one of the 5-mm ports. Although other studies have described the use of the laparoscope and grasping forceps to remove renal stones, the use of the ureteroscope allows access to the periphery of the kidney, and especially lower calyceal stones [6]. Further applications combining endourologic techniques and laparoscopic surgery are currently in development.

The retroperitoneal laparoscopic approach is a minimally invasive alternative to endourologic treatment for a variety of ablative and reconstructive indications for different pathologic conditions. After adequate training, experienced surgeons should be able to use this approach proficiently. Despite the limited working space, direct posterior access to the kidney and renal hilum makes this access attractive, as it allows early renal vessel control.

The laparoscopic approach is an established reconstructive technique in UPJO, and various studies have reported a success rate of more than 95% (up to a mean follow-up of 24 months) [7-11]. In a head-to-head comparison of laparoscopic treatment and percutaneous endopyelotomy for primary UPJO, the success rates were 100 and 92%, respectively [12]. In the present study, concomitant laparoscopic pyelolithotomy with pyeloplasty provided a 100% stone clearance rate, thereby extending the advantage of a minimally invasive approach to all such patients. Similar stone clearance rates have been shown by other studies with equivalent results (80–90%) [7-9,13,14].

Simforoosh [15] and colleagues have reported simultaneous treatment of renal stone and retrocaval ureter with laparoscopy. Similarly, Mugiya [16] and associates reported a case in which a retrocaval ureter and upper ureteric stones were managed during the simultaneous procedure. We believe that the laparoscopic technique should be kept as the first option for the management of retrocaval ureter, even when complicated by renal stones.

The goals of nephropexy—fixation of the kidney at a retroperitoneal position, relief of any urinary obstruction associated with nephroptosis, immobilization of the renal axis, and prevention of tension on the vessels and ureter—are all achieved with our approach.

We found that the retroperitoneal laparoscopy has great sensitivity for detecting a crossing vessel, because it allows the surgeon a view of the UPJ and related vessels in their anatomic position, laterally to medially, by simple elevation of the lower pole of the kidney. Therefore, we amputated the crossing vessel before extracting the stone in one case.

## Conclusion

Based on our experience, retroperitoneal laparoscopic intrasinusal pyelolithotomy is an effective and less invasive alternative for intra-renal pelvic stones, but not for large staghorn stones. For treatment of multiple pelvic calyceal stones, we found that the laparoscope combined with the ureteroscope could grasp calyceal stones through the pelvic incision, which significantly reduced the rate of residual stones. Infectious renal and ureteral stones can be treated in a one-step operation. The advantages of this method include no harm to the nephron, less bleeding, simple manipulation, short hospital stay, and quick postoperative recovery.

For patients with UPJO, retrocaval ureter and nephroptosis, laparoscopic treatment has obvious merits over other minimally invasive methods. We removed obstructions of the urinary tract by performing pyeloplasty, nephropexy, ureteropelvic anastomosis, and amputation of crossing vessels, which effectively reduced the recurrence of stones.

## Abbreviations

UPJO: Ureteropelvic junction obstruction.

## Competing interests

The authors declare that they have no competing interests.

## Authors’ contributions

QL, CJY and WZ conceived of the study, CQ, SQW and PL participated in the design of the study, CQ and QC performed the statistical analysis, CQ, PFS, PCL, ZJH and JT collected the patients data, XXM, XBJ, RJS, JL and QL performed the surgery, CQ and SQW drafted the manuscript. All authors read and approved the final manuscript.

## Pre-publication history

The pre-publication history for this paper can be accessed here:

http://www.biomedcentral.com/1471-2490/14/16/prepub
